# Incidence and Long-term Functional Outcome of Neurologic Disorders in Hospitalized Patients With COVID-19 Infected With Pre-Omicron Variants

**DOI:** 10.1212/WNL.0000000000207534

**Published:** 2023-08-29

**Authors:** Simone Beretta, Viviana Cristillo, Giorgia Camera, Carlo Morotti Colleoni, Gaia Pellitteri, Beatrice Viti, Elisa Bianchi, Stefano Gipponi, Maria Grimoldi, Mariarosaria Valente, Susanna Guttmann, Maria Sofia Cotelli, Pasquale Palumbo, Giorgio Gelosa, Stefano Meletti, Cristina Schenone, Donatella Ottaviani, Massimo Filippi, Andrea Zini, Paola Basilico, Lucia Tancredi, Pietro Cortelli, Massimiliano Braga, Valeria De Giuli, Serenella Servidei, Damiano Paolicelli, Federico Verde, Stefano Caproni, Antonio Pisani, Vincenzina Lo Re, Luca Massacesi, Daria Valeria Roccatagliata, Paolo Manganotti, Daniele Spitaleri, Anna Formenti, Marta Piccoli, Silvia Marino, Paola Polverino, Umberto Aguglia, Raffaele Ornello, Elisabetta Perego, Gabriele Siciliano, Paola Merlo, Marco Capobianco, Leonardo Pantoni, Alessandra Lugaresi, Stefania Angelocola, Anna De Rosa, Maria Sessa, Ettore Beghi, Elio Clemente Agostoni, Salvatore Monaco, Alessandro Padovani, Alberto Priori, Vincenzo Silani, Gioacchino Tedeschi, Carlo Ferrarese, for Neuro-COVID Italy

**Affiliations:** From the Department of Neurology (S.B., C.M.C., C.F.), Fondazione IRCCS San Gerardo dei Tintori, Monza; Department of Medicine and Surgery (S.B., C.M.C., C.F.), University of Milano Bicocca; The Milan Center for Neuroscience (NeuroMI) (S.B., G.G., C.F.); Neurology Unit and Department of Clinical and Experimental Sciences (V.C., S. Gipponi, A. Padovani), University of Brescia; Unit of Neurology and Neurophysiology (G.C., M.G., M.S.), ASST PG23, Bergamo; Santa Maria della Misericordia University Hospital (G.P., M.V.), Udine, Italy; San Marino Neurological Unit (B.V., S. Guttmann), San Marino Hospital; The Mario Negri Institute for Pharmacological Research IRCCS (E. Bianchi, E. Beghi), Milan; Department of Medical Area (DAME) (M.V.), University of Udine; Neurology Unit (M.S.C.), ASST Valcamonica, Esine, Brescia; USL Centro Toscana (P. Palumbo), Neurology Unit, Nuovo Ospedale Santo Stefano, Prato; Department of Neurology and Stroke Unit (G.G., E.C.A.), Niguarda, Milan; Department of Neurology and Department of Clinical Neurophysiology AOU Modena (S. Meletti), University of Modena and Reggio Emilia; Department of Neuroscience, Rehabilitation, Ophtalmology, Genetics, Maternal and Child Health (C.S.), University of Genoa; Ospedale Santa Maria del Carmine di Rovereto (D.O.), Trento; Neurology Unit (M.F.), IRCCS San Raffaele Scientific Institute, Milan; Department of Neurology (A.Z.), Metropolitan Stroke Network, Ospedale Maggiore, Bologna; Department of Neurology (P.B.), Ospedale A. Manzoni ASST Lecco; University of Milan (L.T., L.P., A. Priori); Neurology Unit (L.T., A. Priori), ASST Santi Paolo e Carlo; Aldo Ravelli Center for Neurotechnology and Experimental Brain Therapeutics (L.T.), Milan; IRCCS Institute of Neurological Science of Bologna (P.C.); DIBINEM (P.C.), University of Bologna; UOC Neurology (M.B.), ASST Vimercate; Department of Neurology (V.D.G.), ASST Cremona; Neurophysiopathology Unit, Fondazione Policlinico Universitario A. Gemelli, Rome, Italy; Department of Basic Medical Sciences, Neurosciences and Sense Organs (D.P.), University of Bari; Department of Neurology and Laboratory of Neuroscience (F.V., V.S.), IRCCS Istituto Auxologico Italiano; “Dino Ferrari” Center (F.V., V.S.), Department of Pathophysiology and Transplantation, Università degli Studi di Milano; Neurology Division (S.C.), “S. Maria” University Hospital, Terni; IRCCS Mondino Foundation (A. Pisani), Department of Brain and Behavioral Sciences, University of Pavia; Department of Diagnostic and Therapeutic Services (V.L.R.), IRCCS ISMETT, Palermo; Department of Neurology 2 (L.M.), Careggi University Hospital, Florence; Department of Neurology and Neurosurgery (D.V.R.), ASST di Mantova; Clinical Neurology Unit (P. Manganotti), Cattinara University Hospital, University of Trieste; Department of Neurology (D.L.A.S.), AORN S.Giovanni Moscati, Avellino; Neurology and Stroke Unit (A.F.), Neuroscience Department, ASST-Lecco, Merate; Department of Neurology (M.P.), Ospedale San Filippo Neri, Rome; IRCCS Centro Neurolesi Bonino-Pulejo (S. Marino), Messina; Department of Neurology (P. Polverino), IRCCS Humanitas Research Hospital, Rozzano, Milan; Department of Medical and Surgical Sciences (U.A.), Magna Graecia University of Catanzaro; Department of Biotechnological and Clinical Sciences (R.O.), University of L'Aquila; Department of Neurology (E.P.), Ospedale Valduce, Como; Neurological Clinic (G.S.), University of Pisa; Department of Neurology (P. Merlo), Humanitas Gavazzeni, Bergamo; Department of Neurology (M.C.), S. Luigi Gonzaga Hospital, Orbassano; Ospedale Luigi Sacco (L.P.), Milan; IRCCS Institute of Neurological Science of Bologna (A.L.), UOSI Multiple Sclerosis Rehabilitation; Department of Biomedical Science and Neuromotricity (A.L.), University of Bologna; Department of Neurology (S.A.), Fermo; Department of Neurosciences (A.D.R.), Federico II University, Naples; Neurology Unit and Department of Neurosciences (S. Monaco), University of Verona; IRCCS Fondazione Ospedale Maggiore Policlinico (A. Priori), Milan; and Department of Advanced Medical and Surgical Sciences (G.T.), University of Campania, Naples, Italy.

## Abstract

**Background and Objectives:**

A variety of neurologic disorders have been reported as presentations or complications of coronavirus disease 2019 (COVID-19) infection. The objective of this study was to determine their incidence dynamics and long-term functional outcome.

**Methods:**

The Neuro-COVID Italy study was a multicenter, observational, cohort study with ambispective recruitment and prospective follow-up. Consecutive hospitalized patients presenting new neurologic disorders associated with COVID-19 infection (neuro-COVID), independently from respiratory severity, were systematically screened and actively recruited by neurology specialists in 38 centers in Italy and the Republic of San Marino. The primary outcomes were incidence of neuro-COVID cases during the first 70 weeks of the pandemic (March 2020–June 2021) and long-term functional outcome at 6 months, categorized as full recovery, mild symptoms, disabling symptoms, or death.

**Results:**

Among 52,759 hospitalized patients with COVID-19, 1,865 patients presenting 2,881 new neurologic disorders associated with COVID-19 infection (neuro-COVID) were recruited. The incidence of neuro-COVID cases significantly declined over time, comparing the first 3 pandemic waves (8.4%, 95% CI 7.9–8.9; 5.0%, 95% CI 4.7–5.3; 3.3%, 95% CI 3.0–3.6, respectively; *p* = 0.027). The most frequent neurologic disorders were acute encephalopathy (25.2%), hyposmia-hypogeusia (20.2%), acute ischemic stroke (18.4%), and cognitive impairment (13.7%). The onset of neurologic disorders was more common in the prodromic phase (44.3%) or during the acute respiratory illness (40.9%), except for cognitive impairment whose onset prevailed during recovery (48.4%). A good functional outcome was achieved by most patients with neuro-COVID (64.6%) during follow-up (median 6.7 months), and the proportion of good outcome increased throughout the study period (*r* = 0.29, 95% CI 0.05–0.50; *p* = 0.019). Mild residual symptoms were frequently reported (28.1%) while disabling symptoms were common only in stroke survivors (47.6%).

**Discussion:**

Incidence of COVID-associated neurologic disorders decreased during the prevaccination phase of the pandemic. Long-term functional outcome was favorable in most neuro-COVID disorders, although mild symptoms commonly lasted more than 6 months after infection.

## Introduction

Neurologic presentations or complications of coronavirus disease 2019 (COVID-19) infection have been reported since the first weeks of the pandemic.^1^ A number of studies, mostly performed in hospitalized patients, showed a wide range of newly diagnosed neurologic disorders associated with COVID-19 infection, including self-reported neurologic symptoms such as hyposmia-hypogeusia, cognitive impairment or headache, and clinical neurologic syndromes such as acute encephalopathy, stroke, Guillain-Barré syndrome, status epilepticus, or encephalitis.^2,3^ These “neuro-COVID” disorders have been described with a variable frequency of approximately 8%, ranging from 1% to more than 30% in different studies.^4,5^

Most available studies provided robust data on the frequency of neuro-COVID disorders and in-hospital mortality.^6,7^ Nonetheless, important issues need to be addressed about the incidence of neuro-COVID disorders over time, the effect of treatments or vaccines, and their long-term outcome.

In this study, we investigated the incidence dynamics and long-term neurologic outcomes of neuro-COVID disorders in a large cohort of patients, recruited over the first 70 weeks of the pandemic (March 2020–June 2021).

## Methods

### Study Design

The Neuro-COVID Italy was an investigator-initiated, multicenter, ambispective, cohort study that was conducted at 38 centers in Italy and the Republic of San Marino, exploring the incidence and outcome of neurologic disorders associated with COVID-19 infection. The study design, inclusion criteria, data collection, and monitoring have been published previously.^8^

The study duration was 70 weeks for recruitment and 6 months for follow-up. The retrospective phase of the study referred to the period between March 2020 and September 2020 and was limited to the baseline data collection of retrospective patients while the prospective phase of the study was conducted starting from October 2020 and included both the baseline collection of new patients (up to June 2021) and 6-month follow-up data for all recruited patients (up to December 2021).

### Standard Protocol Approvals and Patient Consents

The study protocol was approved by the Ethics Committee of Istituto Auxologico Italiano (study code 2020_03_26_03) and the Ethics Committees of all participating centers. All participants gave written informed consent, with the exception of deceased patients, according to European Union General Data Protection Regulation and related Italian regulations (art.21 DL 101/2018, art. 110 Privacy Code).

### Patient Population

To be included in the cohort, patients had to be aged older than 18 years and fulfill all the following 3 inclusion criteria:

- a newly diagnosed COVID-19 infection, independent from clinical severity;
- a newly diagnosed neurologic disorder of any kind, whose onset was time-locked to the prodromal phase, acute respiratory phase, or recovery phase of COVID-19 infection; and
- being hospitalized for COVID-19 infection and/or neurologic disorders.

Consecutive patients were exclusively recruited by neurology specialists involved in the study using a systematic multimodal screening approach. All patients who performed at least one neurologic consultation were actively identified and proposed to enter the study. Neurologic consultations included the following sources: direct consultation in Neurology departments, Stroke Units or Neuro-COVID wards; referral from physicians or intensivists working in COVID wards or intensive care units (ICUs); and referral from infectious disease specialists working in dedicated multidisciplinary post-COVID clinics. All patients underwent a full neurologic workup according to their disorders.

### Study Definitions

COVID-19 infection was defined according to the WHO COVID-19 case definition (December 2020),^9^ including both confirmed cases of severe acute respiratory syndrome coronavirus 2 (SARS-CoV-2) infection (positive molecular/antigenic virologic test +/− clinical and epidemiologic criteria) and probable cases of SARS-CoV-2 infection (clinical, radiologic, and epidemiologic criteria, without molecular/antigenic virologic testing). Probable cases represented <10% of the study population and were limited to the first 2 weeks of the pandemic, when molecular/antigenic testing were not routinely available.

COVID waves were defined according to the Italian National Institute of Health data as follows: first COVID wave, March 2020 to June 2020; second COVID wave, July 2020 to January 2021; and third COVID wave, February 2021 to June 2021. The COVID-19 vaccination campaign in Italy started on December 27, 2020.

Acute encephalopathy was defined as a rapidly developing (hours to a few days) pathobiological process in the brain, leading to a clinical presentation of subsyndromal delirium, delirium, or coma, according to a recent consensus of 10 scientific societies.^10^

Encephalitis was defined as altered mental status lasting >24 hours, not attributable to another cause, and the presence of 2 or more of the following criteria: generalized or partial seizures not fully attributable to a preexisting epilepsy, new onset of focal neurologic findings, CSF white blood cell count ≥5 cells/μL, abnormality of brain parenchyma on neuroimaging suggestive of encephalitis, and abnormality on electroencephalography consistent with encephalitis.^11^

Severe respiratory failure was defined as requiring respiratory support with continuous positive airway pressure or mechanical ventilation.

COVID association for subacute and chronic disorders, such as Guillain-Barré syndrome and cognitive impairment, was defined as occurrence within 6 weeks from acute infection, according to a previous study.^4^

### Data Collection

Demographics, comorbidities, clinical characteristics of COVID-19 and neurologic disorders, and outcomes were collected by the centers involved in this study. All data were securely stored in an electronic case report form developed with the Integrated Clinical Trial Environment platform by Advice Pharma, which acted as Contract Research Organization of this study. Data access was protected by username and password according to different profiles, depending on the user.

The number of all hospitalized COVID cases (denominator) during the first, second, and third COVID waves were collected by 22 centers and accounted for 52,759 hospitalized patients with COVID. The proportion of neuro-COVID cases relative to all hospitalized COVID cases was based on the number of neuro-COVID cases recruited in these 22 centers (1,614 patients, which accounted for 86.5% of the total neuro-COVID cohort).

### Study Outcomes

The incidence of neuro-COVID cases was assessed on a weekly basis as the total number of COVID cases presenting a newly diagnosed neurologic disorder of any kind, with onset during the prodromal phase, acute respiratory phase, or recovery phase of COVID-19 infection.

Long-term functional outcome for each neurologic disorder was categorized using a pragmatic, patient-centered, outcome approach as follows: full recovery; mild symptoms, not interfering with activities of daily living (ADLs); and disabling symptoms, interfering with ADLs.

For disorders affecting motor skills (e.g., stroke), “mild symptoms” corresponded to a score of 2 or less of the modified Rankin scale, whereas “disabling symptoms” corresponded to a score of 3 or more. For disorders unaffecting motor skills (e.g., headache), outcome was scored taking into account both the pre-COVID level of functioning and the specific neurologic disorder to be assessed.

For some analyses, outcome was dichotomized into “good neurologic outcome,” including full recovery or mild symptoms, and “poor neurologic outcome,” including disabling symptoms or death. Outcome scoring was performed at 6 months after admission by neurologists involved in this study during a structured outpatient visit or phone call, depending on the patient's condition.

### Statistical Analysis

Descriptive statistics on all available variables were performed in the entire sample of hospitalized patients and separately in patients with specific neurologic disorders. Continuous variables were described with median and interquartile range (IQR) while categorical variables were described with count and percentage. The number and type of newly diagnosed neurologic disorders associated with COVID-19 infection were reported in all hospitalized patients. The number of neuro-COVID cases and the number of specific neurologic disorders occurred by week was calculated and reported over the entire study period. The percentage of neuro-COVID cases over the total of hospitalized COVID cases was calculated considering the entire study period and then separately for first, second, and third waves. Percentages were compared between waves using the Friedman test. Proportion of patients with severe respiratory failure was calculated per week, expressed graphically using Locally Weighted Scatterplot Smoothing and assessed by Pearson correlation coefficient. The time of onset of neurologic disorders was described as count and percentage of patients presenting at least one neurologic disorder during the prodromal phase, the acute respiratory phase, or the recovery phase. The number and percentage of patients presenting specific combinations of neurologic disorders were also examined. All possible combinations of 2, 3, 4, and up to 5 disorders were evaluated. Functional outcome was described with counts and percentages globally and separately by specific neurologic disorders. Functional outcome over time was assessed by Pearson correlation coefficient. Recovery and survival curves were constructed for selected neurologic disorders using the Kaplan-Meier method, with, respectively, resolution or death as event variable and time to resolution or death as time variable. Patients not experiencing the event of interest were censored at the last available follow-up time. Kaplan-Meier curves were compared with the log-rank test.

The association of selected variables (age, sex, preexisting neurologic comorbidities, severe respiratory failure, treatment with anticoagulants, steroids, or remdesivir) with the long-term functional outcome was evaluated using univariable and multivariable logistic regression models, considering dependent variable as the global outcome and separately the outcome of selected neurologic disorders. The selection of variables was made on clinical grounds, including those that were expected to have an impact on neurologic outcome and that were available for all patients. The results were reported as odds ratios (ORs) and adjusted OR with 95% CIs.

Statistical analyses were performed using the SAS statistical package (version 9.4; SAS Institute, Cary, NC). The significance level was set to 0.05. Missing data were handled using the list-wise deletion method.

### Data Availability

Investigators may request access to anonymized individual patient data and redacted trial documents including raw data sets, analysis-ready data sets, trial protocols, annotated case report form, statistical analysis plan, and data set specifications. Before the use of data, proposals need to be approved by the Neuro-COVID Italy Steering Committee, and a signed data sharing agreement will then be approved.

## Results

### Study Population

The cohort consisted of 1,865 patients with neuro-COVID, presenting 2,881 neurologic disorders (Tables 1 and 2). The median age was 68 years (IQR 57–78), with a male predominance (60.2%). Most patients had systemic comorbidities (82.2%), particularly hypertension (49.1%), diabetes (19.3%), and chronic heart disease (10.8%). Approximately one-fifth of patients had preexisting chronic neurologic disorders, particularly cerebrovascular diseases (12.0%) and neurodegenerative diseases (10.1%). For most analyses, 4 major self-reported neurologic symptoms (hyposmia-hypogeusia, cognitive impairment, headache, and dizziness) and 6 major clinical neurologic syndromes (acute encephalopathy, acute ischemic stroke, Guillain Barrè syndrome, seizures and status epilepticus, encephalitis, and hemorrhagic stroke) were considered. A complete list of neurologic disorders is shown in eTable 1 (links.lww.com/WNL/C963).

**Table 1:**
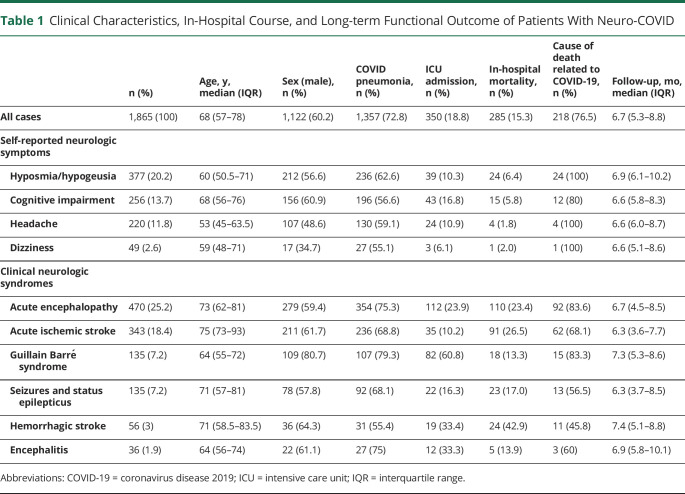
Clinical Characteristics, In-Hospital Course, and Long-term Functional Outcome of Patients With Neuro-COVID

**Table 2:**
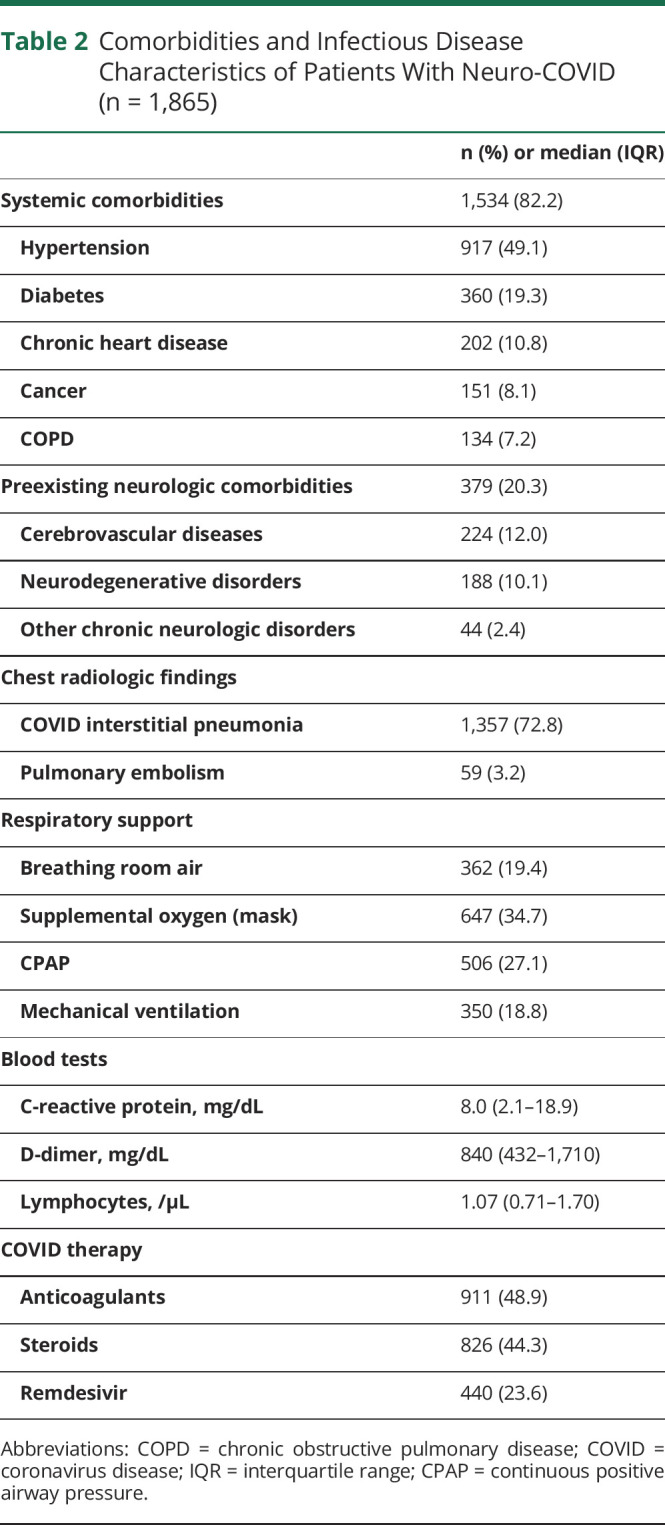
Comorbidities and Infectious Disease Characteristics of Patients With Neuro-COVID (n = 1,865)

### Intrapandemic Incidence Dynamics

**Figure 1:**
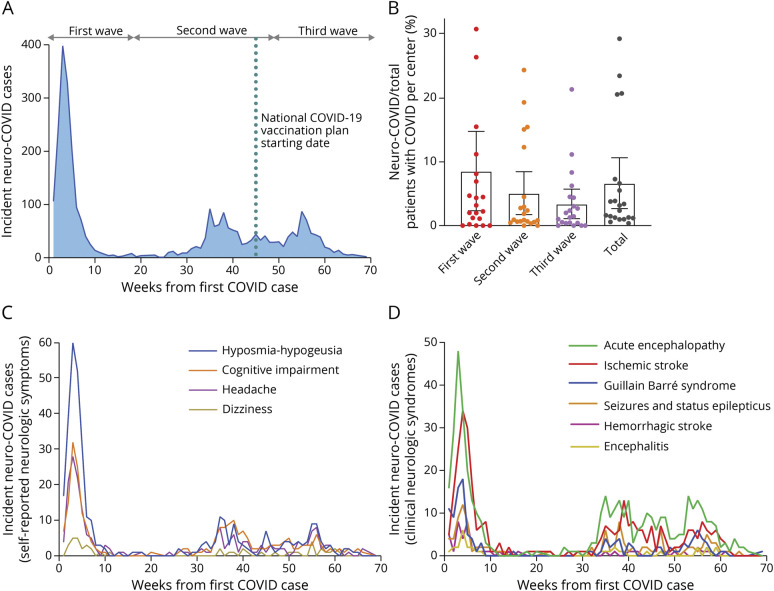
Incidence Dynamics of Neuro-COVID Disorders Intrapandemic incidence of neuro-COVID cases (A) and their proportion relative to all hospitalized COVID cases (B; each dot represents a recruiting center). Incidence is represented separately for selected self-reported neurologic symptoms (C) and clinical neurologic syndromes (D). COVID = coronavirus disease.

**Figure 2:**
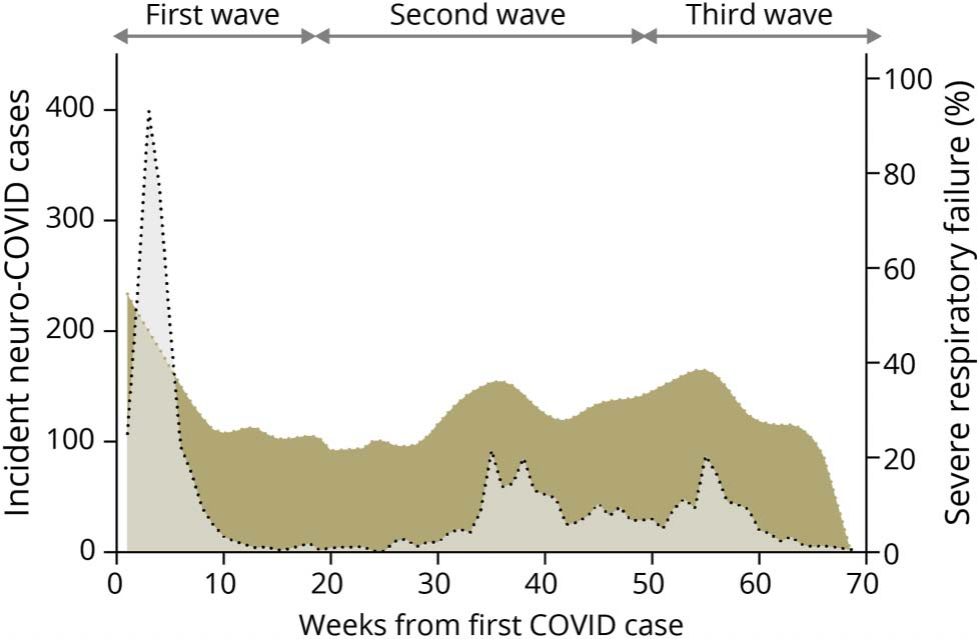
COVID-Related Severe Respiratory Failure and Incidence of Neuro-COVID Disorders LOWESS was used to represent graphically the proportion of patients with severe respiratory failure, per week (colored area). Incident neuro-COVID cases are represented shaded in gray. COVID = coronavirus disease; LOWESS = locally weighted scatterplot smoothing.

The incidence of neuro-COVID cases showed a highly dynamic pattern during the first 70 weeks of the pandemic, which mostly included the prevaccination era (Figure 1, A and B). Both the absolute number of new neuro-COVID cases and the percentage of new neuro-COVID cases over a total of 52,759 hospitalized COVID cases showed a peak during the first wave (8.4%, 95% CI 7.9–8.9), followed by a significant reduction during the following 2 waves (5.0%, 95% CI 4.7–5.3, and 3.3%, 95% CI 3.0–3.6, respectively; *p* = 0.027). This downward trend did not reflect the number of total hospitalized COVID cases, which actually showed an increase in both the second (+61.0%) and third (+23.5%) waves, compared with the first wave. A similar incidence dynamics was observed for the 4 self-reported neurologic symptoms and the 6 clinical neurologic syndromes, considered separately (Figure 1, C and D). Differently from the incidence of neuro-COVID disorders, which declined over time (*r* = −0.364, 95% CI −0.56 to −0.13; *p* = 0.003), the proportion of patients with neuro-COVID with severe respiratory failure did not change during the study period (*r* = −0.053, 95% CI −0.29 to 0.19; *p* = 0.674; Figure 2).

### Frequency, Onset, and Clustering

**Figure 3:**
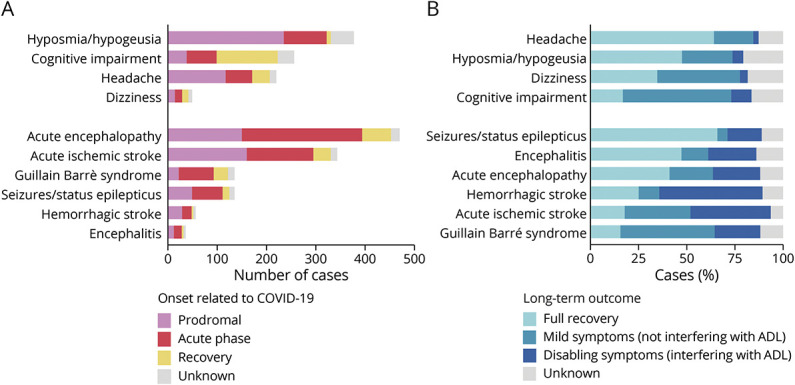
Frequency, Onset, and Long-term Functional Outcome of Neuro-COVID Disorders Frequency and onset are represented as absolute number of cases (A) while functional outcome categories are represented as % of cases (B). ADL = activities of daily living; COVID = coronavirus disease.

The 2 most frequent self-reported neurologic symptoms were hyposmia-hypogeusia (20.2%) and cognitive impairment (13.7%), followed by headache (11.8%) and dizziness (2.6%). The 2 most frequent clinical neurologic syndromes were acute encephalopathy (25.2%) and acute ischemic stroke (18.4%), followed by Guillain Barré syndrome (7.2%) and seizures and status epilepticus (7.2%) while hemorrhagic stroke (3%) and encephalitis (1.9%) were relatively rare (Figure 3A). The onset of neurologic disorders was more frequent during the prodromal phase of COVID for hyposmia-hypogeusia (62.3%) and headache (53.2%); during the acute respiratory phase for acute encephalopathy (51.2%) and Guillain Barré syndrome (52.5%); and during recovery from COVID for cognitive impairment (48.4%). The onset of acute ischemic stroke, seizures and status epilepticus, hemorrhagic stroke, and encephalitis occurred in either the prodromal or the acute respiratory phase, without a clear prevalence (42.0% vs 40.9%, respectively, considering these 4 disorders as a single group). No consistent (>5%) association between 2 or more neurologic disorders was observed in our population (eFigure 1, links.lww.com/WNL/C963). Major diagnostic tests of selected neuro-COVID disorders are shown in eTable 2.

### In-Hospital Course

Most patients with neuro-COVID developed a radiologically defined interstitial pneumonia (72.7%) and required some form of respiratory support (80.6%), with 45.9% of cases developing severe respiratory failure and 18.8% requiring ICU admission (Tables 1 and 2). Overall, in-hospital mortality was 15.3% and in most patients (76.5%) was deemed to be related to COVID infection, as opposed to neurologic disorders, albeit with some variability. In-hospital mortality for acute encephalopathy and acute ischemic stroke occurred approximately in one-fourth of cases (23.4% and 26.5%, respectively), although the cause of death was still deemed to be COVID-related in most of these patients (83.6% and 68.1%, respectively). By contrast, in-hospital mortality for intracerebral hemorrhage was the highest (42.9%) and the lowest attributable to COVID (45.9%). In-hospital mortality for Guillain Barré syndrome, seizures/status epilepticus, and encephalitis was approximately 10%–15%, which is not different from what was expected from these disorders, independently from COVID association.

### Long-term Functional Outcome

Long-term outcome data were available for 1,601 (85.8%) patients with a median follow-up duration of 6.7 months (IQR 5.3–8.8) (Figure 3B). Overall, a good functional outcome (full recovery or mild symptoms) occurred in 1,206 (64.6%) patients. A subgroup analysis of the working age population (age 18–64 years) showed a higher proportion of good functional outcome (72.5%), although full recovery was achieved in 31.4% and mild symptoms persisted in 41.1% (eTable 3, links.lww.com/WNL/C963).

Long-term disabling symptoms were reported by a small fraction (5.7%) of patients with any self-reported neurologic symptoms. Full recovery was more frequent in patients with headache (64.1%) and hyposmia-hypogeusia (47.5%) while persistence of mild symptoms was more common in patients with cognitive impairment (56.2%) and dizziness (42.8%). Long-term sequelae were more severe for clinical neurologic syndromes, which presented long-term disability in approximately half of cases for cerebrovascular diseases (cerebral hemorrhage 53.5%; acute ischemic stroke 41.6%) and in roughly one-fourth of cases for encephalitis (25%), acute encephalopathy (24.4%), and Guillain Barrè syndrome (23.7%) while patients with seizure and status epilepticus exhibited a slightly lower proportion of persistent disabling symptoms (17.7%) and the highest chance of full recovery (65.9%).

**Figure 4:**
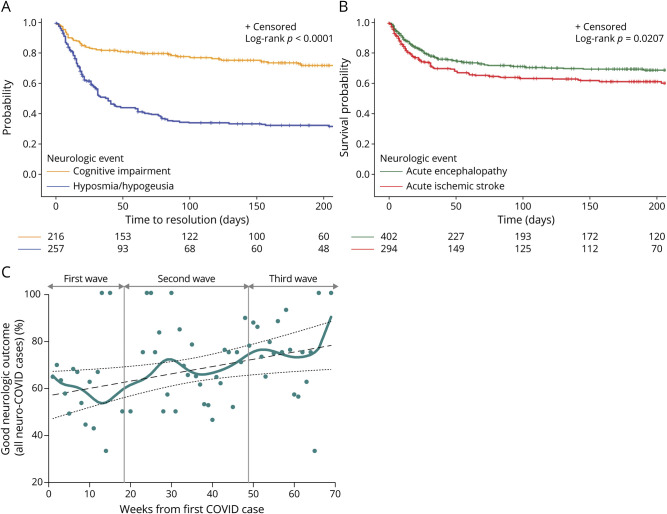
Recovery and Survival Curves of Selected Neuro-COVID Disorders Recovery curves of cognitive impairment and hyposmia-hypogeusia (A) and survival curves of acute encephalopathy and acute ischemic stroke (B). Dynamics of good functional outcome is shown for all recruited cases (C). COVID = coronavirus disease.

Recovery curves (Figure 4A and eFigure 2, links.lww.com/WNL/C963) showed that time to resolution was highly variable, and a considerable proportion of patients with any self-reported symptoms have not yet fully recovered after 6 months. Particularly, incomplete recovery was considerably more common for cognitive impairment compared with hyposmia-hypogeusia (approximately 70% vs 40%; log-rank *p* < 0.0001). Survival curves (Figure 4B and eFigure 3) showed that mortality prevailed in the first several weeks after admission and was slightly higher for acute ischemic stroke compared with acute encephalopathy (approximately 40% vs 30%; log-rank *p* = 0.020). Severe respiratory failure in the acute phase did not affect time to recovery for hyposmia-hypogeusia and cognitive impairment or survival for acute encephalopathy. Conversely, severe respiratory failure in the acute phase largely reduced survival in patients with acute ischemic stroke (eFigure 4).

Overall, the proportion of patients with neuro-COVID achieving a good functional outcome moderately improved over the 3 pandemic waves (Pearson *r* = 0.29, 95% CI 0.05–0.50; *p* = 0.019; Figure 4C). Multivariable analysis showed that age and severe respiratory failure independently predicted a poor functional outcome while treatment with remdesivir independently predicted a good functional outcome (Table 3; details on selected disorders are reported in eTable 4, links.lww.com/WNL/C963).

**Table 3:**
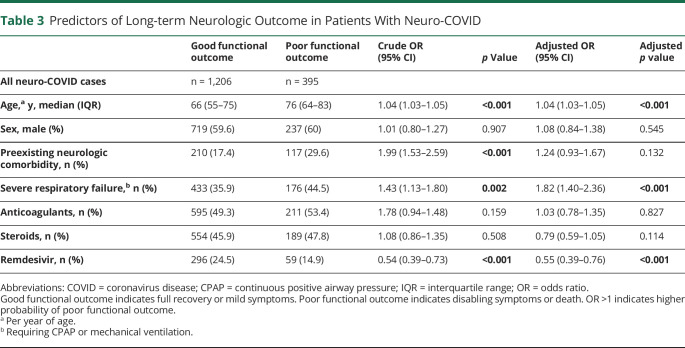
Predictors of Long-term Neurologic Outcome in Patients With Neuro-COVID

## Discussion

Neurologic disorders associated with COVID-19 infection, collectively known as “neuro-COVID,” are among the most alarming, controversial, and least understood aspects of the current pandemic.^12^ Since 2020, efforts have been made to define causality^13^ and the strength of evidence for an association^14^ as well as frequency and in-hospital mortality of neuro-COVID disorders.^6,7^ However, no evidence is available on their incidence dynamics, onset, and long-term outcome.

In the Neuro-COVID Italy study, we report detailed, long-term data from a large cohort of patients with neuro-COVID from the first 1.8 years of the pandemic (1.3-year recruitment + 0.5-year follow-up), mostly related to the original SARS-CoV-2 virus (Wuhan-Hu-1) and its variants Alpha and Delta.^15^

Our findings clearly indicate that the incidence of all major neuro-COVID disorders declined and long-term functional outcome improved during the first 3 pandemic waves. We may hypothesize 2 reasons underlying this phenomenon. First, genomic variations of the SARS-CoV-2 virus, which occurred between the COVID waves, reduced its pathogenic potential for neurologic disorders. This scenario could be similar to what was observed for the multisystem inflammatory syndrome in children, whose rate declined passing from Alpha to Delta and, even more, to Omicron variants.^16^ Second, overall management of patients with COVID-19 improved over time (early diagnosis, home-based treatment, and inpatient care) and contributed to prevent some neurologic disorders. According to our data, the reduction in the incidence of neuro-COVID cases was not associated with changes in the rate of severe respiratory failure. Interestingly, a recent study reported fewer COVID-19 neurologic complications in hospitalized patients treated with dexamethasone, remdesivir, or both drugs combined.^17^

The results of this study substantially reflect the prevaccination era because 75.4% of recruited neuro-COVID cases occurred before the start of the national COVID vaccine campaign (December 27, 2020) and the remaining 24.6% occurred in the January–June 2021 period, when only 31.8% of the total population had received the primary vaccination course.^15^ Although data on vaccination status was not collected, the estimated proportion of vaccinated patients hypothetically enrolled in the Neuro-COVID Italy study falls below 5%, after crossing the accrual data with the demographic reports of the Italian National Institute of Health.^15^ Thus, a major effect of the vaccines is unlikely to be responsible for the reduction of COVID-associated neurologic disorders observed in this study.

Our findings are apparently in contrast with a recent large retrospective study, which reported a similar risk of receiving a neurologic or psychiatric diagnosis after Alpha, Delta, and Omicron variants. However, major differences in methodology may explain these discrepancies, in particular the use of health records, matching with a cohort of patients with other respiratory infections, inclusion of both adults and children from either hospital or community setting, and COVID association projected up to 2 years after infection.^18^

Collectively, long-term outcome of neuro-COVID disorders proved to be rather favorable. However, persistence of mild symptoms for many weeks or months was common for all self-reported symptoms, particularly cognitive impairment, in agreement with previous reports.^19^ Long-term disability and death were more frequent than expected only for COVID-associated cerebrovascular diseases and acute encephalopathy, compared with other known causes of the same disorders, in accordance with previous studies.^20-22^

Diagnosis, treatment, data collection, and follow-up visits or calls were exclusively performed by neurologists who were involved in patient's management from hospital admission or were referred the patient by COVID physicians, intensivists, or infectious disease specialists at any time during the study period. This neurologist-centered approach of patient ascertainment had both advantages and limitations. A major advantage was high standards of diagnostic accuracy and disease management, which was guaranteed by specialized medical professionals, with direct consequences on disease definitions, data collection, and patient outcome. A major limitation was that some patients with minor neurologic symptoms, such as mild confusion or transient headache, may have been missed if they had never been seen by a neurologist. However, these minor neuro-COVID symptoms were not the focus of our work, which was dedicated to serious or long-lasting neurologic disorders associated with COVID infection. Another possible source of under-recruitment is related to the first few weeks of the pandemic in Italy, when the hospital system was unprepared and overstressed, and some patients with overwhelming respiratory symptoms may have died before diagnosing any concomitant neurologic disorders. We recognize that this (expected) ascertainment bias is the most likely explanation for the slightly lower proportion of neuro-COVID cases in our cohort, compared with a recent meta-analysis of individual patient data (6.6% vs 7.8%).^4^

Neurologic disorders associated with COVID-19 infection progressively decreased during the early (pre-Omicron, prevaccination) phase of the pandemic. Long-term functional outcome was favorable in most neuro-COVID disorders and improved over time, although mild symptoms commonly lasted more than 6 months after infection. Although COVID-19 infection evolved quickly, our results could be potentially relevant for many world regions that did not apply a massive vaccination campaign and also for new coronaviruses that may arise in the future. Further studies are needed to assess the aftermath of the Omicron variant and vaccines, the socioeconomic impact of neuro-COVID disorders, and the risk of developing neurologic diseases long after COVID-19 infection.

## Glossary

ADL: activities of daily living
COVID-19: coronavirus disease 2019
ICU: intensive care unit
IQR: interquartile range
OR: odds ratio
SARS-CoV-2: severe acute respiratory syndrome coronavirus 2
